# Temporal association between serious bleeding and immunization: vitamin K deficiency as main causative factor

**DOI:** 10.1186/s12887-020-1983-8

**Published:** 2020-02-21

**Authors:** Susi Susanah, Eddy Fadlyana, Meita Dhamayanti, Rodman Tarigan, Eko Fuji Ariyanto, Yunisa Pamela, Yuzar I. B. Ismoetoto, Rita Verita Sri, Monika Hasna, Kusnandi Rusmil

**Affiliations:** 1 Department of Child Health, Dr. Hasan Sadikin General Hospital/Faculty of Medicine, Universitas Padjadjaran, Bandung, Indonesia; 2 Department of Child Health, Hematology-Oncology Division, Dr. Hasan Sadikin General Hospital/Faculty of Medicine, Universitas Padjadjaran, Bandung, Indonesia; 3 Department of Biomedical Sciences, Division of Biochemistry and Molecular Biology, Faculty of Medicine, Universitas Padjadjaran, Bandung, Indonesia; 4 West Java Provincial Committee of AEFI, Regional Health Office of West Java Province, Bandung, Indonesia; 5 West Java Provincial Committee of AEFI, District Health Office of Bandung City, West Java Province, Bandung, Indonesia; 6 Chief of West Java Provincial Committee of AEFI, Regional Health Office of West Java Province, Bandung, Indonesia

**Keywords:** AEFI, Bleeding, Children, Immunization, Vitamin K deficiency bleeding

## Abstract

**Background:**

Bleeding as an adverse event following immunization (AEFI) that is rarely reported in children, although it can be a parental concern. Bleeding episodes ranging in severity from mild to severe and defined as any external and/or internal bleeding can be caused by acquired or hereditary disorders. This study analyzes whether bleeding episodes in children that were recorded as AEFIs are causally associated with immunization and elaborates their etiology.

**Methods:**

A cross-sectional study of 388 AEFI cases in children from West Java Provincial Committee in Indonesia confirmed by case findings from 2000 until 2017.

**Results:**

Of the total number of cases studied, 55 (14%) involved children aged 5 days to 12 years who presented with bleeding and were referred to a provincial hospital. Analysis revealed that 32 cases were most likely caused by acquired prothrombin complex deficiency (APCD) and 30 of these APCD cases were strongly suspected to be manifestations of vitamin K deficiency bleeding (VKDB). All VKDB subjects were aged 5 days to 3 months without a history of administration of prophylactic vitamin K. When a World Health Organization classification was used, most bleeding cases in this study became coincidental events with a temporal association with immunization. A causality assessment suggested that these cases were causally unrelated.

**Conclusion:**

Most cases of bleeding reported as an AEFI were found to be VKDB, which is considered a coincidental event following immunization with a temporal association, and an unrelated category based on the results of a causality assessment. Vitamin K should be administered to all newborns as a prophylactic and AEFI surveillance should be improved based on the low numbers of AEFI reported in Indonesia.

## Background

Bleeding in children is often a great concern for parents and physicians, as it is frequently suggestive of an underlying disorder [1–4]. Bleeding can occur spontaneously following medical procedures such as a surgery, circumcision, or injection [3, 4]. When bleeding takes place following administration of immunization, it is sometimes considered an adverse event following immunization (AEFI), which is defined by WHO as any unfavorable sign, symptom, laboratory result, or disease that occurrs after immunization, although it may not necessarily be caused by the vaccination [5].

Although they have not been widely reported, bleeding symptoms have been reported as AEFIs. Immune thrombocytopenia purpura (ITP) was reported and reviewed as an AEFI following administration of measles-mumps-rubella, hepatitis B, and hepatitis A vaccinations [6, 7]. A study by Hamiel et al. recounted bleeding symptoms as a manifestation of thrombocytopenia in a patient receiving the influenza vaccine [8]. Susanah et al. reported bleeding manifestations as AEFI cases in West Java Province, Indonesia, most due to vitamin K deficiency, followed by cases caused by underlying diseases, either hereditary hemostasis disorders such as hemophilia, or acquired hemostasis disorders such as dengue hemorrhagic fever [9].

Because vaccines are given to healthy individuals, a higher standard of safety is generally expected when compared with other medical interventions [5, 10]. Fear of AEFIs can deter people from getting an immunization; therefore, every case of AEFI must be investigated thoroughly to maintain public trust and improve immunization coverage [5, 10]. Assessment of AEFIs is crucial to determining any causal association with the preceding immunization, or whether it is just a temporal coincidence [5, 10, 11]. In this study, we analyzed AEFI cases, in particular bleeding symptoms, that occurred following immunization.

## Methods

### Study design and participants

A descriptive cross-sectional study was conducted from 2000 to 2017 in West Java Province, Indonesia, involving the West Java Provincial AEFI Committee, Dr. Hasan Sadikin General Hospital (the only provincial referral hospital in West Java), regional hospitals, and primary health care facilities.

The study included 388 subjects who reported experiencing an AEFI to the West Java Provincial AEFI Committee from 2000 to 2017. Of those subjects, 55 with bleeding manifestations were further examined.

### Characteristic of participants

Surveillance of AEFI cases in Indonesia has proven challenging, and the Indonesian AEFI reporting system was initiated by the Ministry of Health Republic of Indonesia in 1998 through the establishment of the National AEFI Committee, which has been tasked with controlling and managing AEFI cases. The surveillance system for AEFIs in Indonesia applies a case-finding method by receiving AEFI case reports from the community and/or health workers in primary or secondary health care facilities. The reports are then forwarded to district health offices to confirm severe AEFIs. After confirmation, these cases are reported to the regional health office, which then notifies the provincial and national AEFI committees. Assessment and investigation of severe AEFIs are performed by professional health workers. Minor AEFIs, which are not serious and do not threaten the health of vaccine recipients, are treated by primary or secondary health care facilities, while severe AEFIs (serious adverse events) that may result in death, cause life-threatening hospitalization, prolong current hospitalization, result in a persistent or significant disability/incapacity, or lead to a congenital birth defect are referred to and treated at the tertiary health care level [5, 12].

In West Java Province, the provincial AEFI committee was established in 2004 as a collaboration between the West Java Health Office and experts from Dr. Hasan Sadikin General Hospital. Before the committee was established, the AEFI surveillance system consisted of reporting severe AEFI cases to the respective district health office for submission to the provincial health office, followed by an audit by health care professionals at Dr. Hasan Sadikin General Hospital, where the patients received proper management.

This study began with identifying and collecting the medical records of patients with a reported AEFI referred to Dr. Hasan Sadikin General Hospital based on a West Java Provincial AEFI Committee report. Subjects of this study came from various areas of West Java Province. Only medical records of patients who had received an immunization in the month prior to the adverse event were included.

**Fig. 1:**
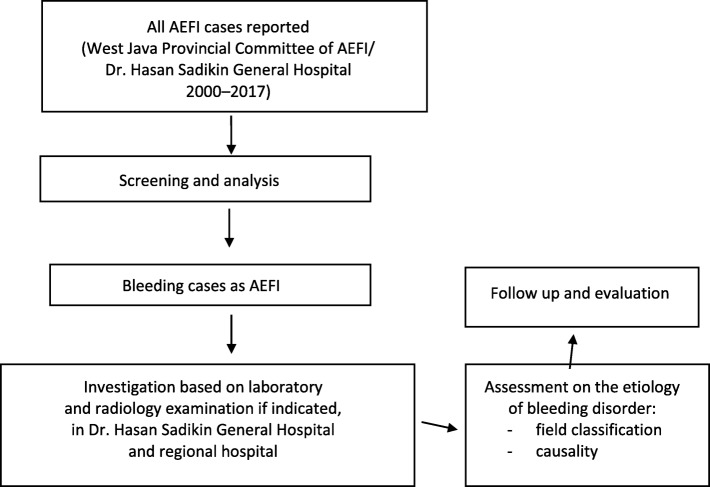
Workflow to determine the etiology of bleeding disorders in AEFIs

To determine the etiology of bleeding disorders in AEFI, the West Java Provincial AEFI Committee (before 2004, the West Java Health Office and experts from Dr. Hasan Sadikin General Hospital team) used an algorithm recommended by the Indonesian Ministry of Health in its regulation of immunization implementation, as summarized in Fig. 1 [12]. This algorithm begins with a comprehensive assessment of the association between adverse events and immunization. The cases are then classified as a vaccine reaction, program error, coincidental, injection reaction, or unknown using WHO’s field classification system [5]. WHO categories were then used to classify the cases into a more specific AEFI categories [5].

Based on WHO Immunization Safety Surveilance Guidelines, AEFIs are determined by field classification and causal assessments. The field classification consists of 5 groups. In the first group, a vaccine reaction is an event caused or precipitated by the vaccine when given correctly; that is. a reaction caused by the inherent properties of the vaccine. The second group consists of events caused by an error in vaccine preparation, handling, or administration, which is considered a program error. The third group covers events that happen after immunization, but are not caused by the vaccine, or a chance association, referred to as a coincidental event. The fourth group contains injection reactions triggered by anxiety about, or pain from, the injection itself, rather than the vaccine’s contents. The last group consists of events with causes that cannot be determined, which are termed unknown [5].

To assess and determine the causality of AEFI cases, WHO has classified AEFI cases into several causality assessment categories of very likely/certain, probable, possible, unlikely, and unrelated. A clinical event with a plausible temporal relationship to vaccine administration that cannot be explained by concurrent disease or other drugs or chemicals is defined as very likely/certain, while probable cases are defined as clinical events with a reasonable temporal relationship with vaccine administration that are unlikely to be attributable to concurrent disease or other drugs or chemicals. Possible cases are described as clinical events with a reasonable temporal relationship with vaccine administration, but which could also be explained by a concurrent disease or other drugs or chemicals. The unlikely category refers to clinical events where the temporal relationship with vaccine administration makes a causal connection improbable, but could be plausibly explained by an underlying disease, other drugs, or chemicals. The unrelated category is applied to a clinical event with an incompatible temporal relationship that could be explained by an underlying disease or other drugs or chemicals, and the unclassifiable category is defined as a clinical event with insufficient information to permit assessment and identification of the cause [5].

In our study, the cases were investigated by analyzing data obtained from AEFI reports and medical records, including age, chief complaint, prior immunization, history of bleeding disorder, physical examination, laboratory work-up, and outcome of subjects. Patients were considered to have experienced bleeding if they suffered from any kind of bleeding manifestation, generally cutaneous (ptechiae, purpura), mucocutaneous (ecchymosis, epistaxis, gum bleeding), and/or internal bleeding, such as intracranial bleeding manifested as convulsion and/or unconsciousness [1–4]. Patients were then analyzed further to determine the underlying bleeding disorder and its etiology.

A diagnosis of a bleeding disorder was made based on bleeding manifestations and laboratory examination results consisting of a complete blood count; hemostasis status that included platelet count, prothrombin time (PT), activated partial thromboplastin time (aPTT), PTT substitution test, factor assay, fibrinogen level, thrombin time (TT); and other supporting examination, such as a computed tomography scan, if indicated [4]. Vitamin K deficiency bleeding (VKDB), one of the etiologies of bleeding disorders, was diagnosed based on a hemostatic analysis showing prolonged PT and aPTT, a PTT substitution test result indicating partially or completely decreased vitamin K–dependent coagulation factors (II, VII, IX, X) with normal fibrinogen levels, and a TT that responded to vitamin K administration [13]. Undercarboxylated prothrombin (factor II) or other proteins induced by vitamin K absence (PIVKA) were not measured as the related tests were not available. VKDB was classified as early (within 24 h), classical (after 2 to 7 days), or late-onset (between 8 days and 6 months after) [13–16].

The presence of liver disease was identified through history-taking, clinical manifestation, and liver function tests, including measurements of aspartate aminotransferase, alanine aminotransferase, serum totals, and direct bilirubin levels [4].

Patients identified as experiencing bleeding as an AEFI during the study were diagnosed by the team, and then treated by a team of pediatricians (led by pediatric hematologists), an immunization task force, and other medical experts at Dr. Hasan Sadikin General Hospital in a collaborative effort.

### Statistical analysis

Data were processed and analyzed using Excel 2010 for Windows and presented in proportion. This study was reviewed and approved by the Ethical Committee of Dr. Hasan Sadikin General Hospital and conducted in accordance with the World Medical Association Declaration of Helsinki’s Ethical Principles for Medical Research Involving Human Subjects (1983 onwards). Informed consent was obtained from the subjects’ parents.

## Results

All AEFI cases were reported by primary or secondary health care facilities based on individual reports from all districts of West Java to the West Java AEFI Committee from 2000 to 2017. Of these, 388 cases included all data necessary for analysis, with 55 cases reported bleeding manifestation.

Bleeding was the fourth most common manifestation of AEFIs after fever (*n* = 88, 22.7%), seizure (*n* = 74, 19.1%), and edema at the site of injection (*n* = 56, 14.4%). Bleeding, such as ecchymosis or hematomas, with serious manifestation with or without seizure, decreased consciousness, and anemia, was seen in 55 (14.2%) AEFI cases. Altered levels of consciousness with a cause other than bleeding occurred in 26 (6.7%) subjects, while irritable patients were identified in 7 (1.8%) cases. Four (1.0%) patients had erythema and 2 (0.5%) of those experienced anaphylactic shock. A total of 76 (19.6%) cases grouped as “others” involved fatigue, gastrointestinal problems, persistent crying, and syncope.

Characteristics of subjects with AEFI in the form of bleeding (*n* = 55 cases) are presented in Table 1. Subjects’ ages ranged between 5 days and 12 years, with the majority of the subjects (80.0%) being male. Most (40.0%) of the reported bleeding cases were preceded by injection of hepatitis B_0_ (Hep B_0_) vaccine. Hep B_0_ is the first hepatitis B vaccination given during 12 h after birth [12].

**Table 1:** Characteristic of subjects with AEFI (*n* = 388)

Characteristics		Bleeding (*n* = 55)		No Bleeding (*n* = 333)	
Age	0-1 year	45	(81.8%)	313	(94.0%)
	1–2 year	6	(10.9%)	15	(4.5%)
	> 2 year	4	(7.3%)	5	(1.5%)
Gender	Male	44	(80.0%)	196	(58.9%)
	Female	11	(20.0%)	137	(41.1%)
Vaccination	Hep B_0_	22	(40.0%)	68	(20.4%)
	BCG	12	(21.8%)	25	(7.5%)
	Polio	0	0	14	(4.2%)
	DTP	3	(5.4%)	36	(10.8%)
	DTP-HB	4	(7.3%)	91	(27.4%)
	DTP-HB-Hib	9	(16.4%)	72	(21.6%)
	Measles	4	(7.3%)	22	(6.6%)
	Td	1	(1.8%)	5	(1.5%)
AEFI classification	Vaccine reaction	0	0	149	(44.8%)
	Program error	4	(7.3%)	45	(13.5%)
	Coincidence	41	(74.5%)	56	(16.8%)
	Injection reaction	0	0	58	(17.4%)
	Unknown	10	(18.2%)	25	(7.5%)
Outcomes	Recovered	37	(67.3%)	298	(89.5%)
	Sequelae	2	(3.6%)	12	(3.6%)
	Died	16	(29.1%)	23	(6.9%)

As shown in Table 1, most cases of bleeding were consistent with acquired prothrombin complex deficiency (APCD) (32/55) and these cases were classified as severe AEFI reactions. From 55 bleeding cases, 41 were deemed coincidental events following immunization.

Other than APCD, 2 cases of hemophilia and 2 of acute leukemia, as well as cases of Henoch-Schonlein purpura and von Willebrand disease, each seen in a single case, were identified after diphtheria-tetanus-pertussis vaccinations. A case of cytomegalovirus infection was identified after a Hep B_0_ vaccination and 1 proven case of dengue hemorrhagic fever with epistaxis, fever, and thrombocytopenia manifestations was seen after tetanus-diphtheria vaccinations in school. Another case with ITP was identified after a measles vaccination, with an apparent causal association. Four cases with massive ecchymoses and/or hematomas at the site of the injection with an abscess as the initial complaint were identified as program errors. The remaining 10 cases involved an unknown etiology.

The 30 APCD cases were diagnosed as VKDB consisting of classical and late-onset VKDB and affected male babies primarily. Most VKDB cases were temporally associated with a hepatitis B vaccine. All patients with VKDB had no prior history of prophylactic vitamin K injection, were exclusively breast-fed infants, and were healthy when they received the injection. Most suffered from intracranial bleeding, with and without severe anemia. (Table 2).

**Table 2:** VKDB-related coincidental AEFIs by type of vaccination (*n* = 30)

Characteristic	Total	BCG (*n* = 5)	HB_0_ (*n* = 20)	DTP-HB (*n* = 5)
Age	5 days-3 months			
Onset	0	0	0	0
Early (< 24 h)	10/30	1	7	2
Classical (day 2–7)	20/30	4	13	3
Late (day 8–6 months)				
Gender				
Male	19/30	3	12	4
Female	11/30	2	8	1
Exclusively breast-fed infant	30/30	5	20	5
Usage of broad spectrum antibiotic	–	0	0	0
Intracranial bleeding	18/30	4	10	4
Severe Anemia	11/30	2	6	3
Outcome				
Life	14/30	3	9	2
Death	16/30	2	11	3

All VKDB-related AEFI cases appeared at the age of 5 days to 3 months, with the male gender predominating (19/30). All subjects were exclusively breast-fed infants and none had used broad-spectrum antibiotics. Eighteen of the 30 VKDB cases demonstrated intracranial bleeding and 11 suffered from severe anemia (Hb < 7 g/dL) [17]. More than 50% of these patients died (16/30).

The incidence of VKDB-related AEFI was reported every year during the study period and appeared to decrease by year. In 2004, 2005, and 2008, 5 cases were reported per year. In 2006, no VKDB-related AEFI was reported. Two cases were reported in 2000–2003, 2007, and 2012–2014. In 2009 and 2011, 3 cases were reported each year. One case was reported in 2010.

## Discussion

Immunization safety is crucial issue ensure the efficacy of a national vaccine-preventable disease control program. To monitor immunization safety, AEFI surveillance provides an effective way to monitor safety and contributes to the credibility of an immunization program that will eventually win public trust. Assessment and investigation of AEFI cases are needed to distinguish coincidental events from true AEFIs and correct program errors [5].

In this study, the frequency of AEFI cases appears to low. The incidence of AEFI associated with the expanded program immunization (EPI) in Indonesia during 1998–2002, as reported to the National AEFI Committee, was 182, with most cases categorized as a coincidence (84.2%) and the remaining cases categorized as a vaccine reaction (39.6%) [18]. The low number of cases may be a product of the case-finding method used by the Indonesian surveillance system, which depends on reports from health care facilities and communities, and which may have led to many unreported AEFI cases. All case information was obtained from health care facilities.

Most AEFIs with bleeding manifestation in this study (41/55) were considered coincidental due to an absence of evidence of causation, and the results of a causality assessment suggested unrelated categories [5, 19]. It is possible that temporal associations are often conflated with an immunization-related cause. These purely temporal associations are unavoidable. Some cases proved to be program errors caused by inappropriate injection techniques, which are preventable [5].

All cases with bleeding manifestation suspected to be an AEFI were referred to the regional hospital or Dr. Hasan Sadikin General Hospital as a severe condition involving seizure, unconsciousness, and severe anemia requiring hospitalization. These cases were considered serious adverse events [5].

The etiology of severe bleeding as a manifestation of an AEFI in this study was mainly due to VKBD-related APCD. All cases had no history of prophylactic vitamin K administration during the neonatal period. VKDB diagnoses were established clinically, and laboratory results suggested that the condition was due to deficiency of vitamin K, with partially or completely decreased factors II, VII, IX, and X producing a hypocoagulable state that responded to vitamin K administration [13–16]. This study did not measure undercarboxylated prothrombin (factor II) or PIVKA to confirm VKDB as the relevant tests were not available. This measurement aims to exclude liver disease and disseminated intravascular coagulation (DIC), two conditions that can be ruled out by clinical manifestations and laboratory tests. Liver diseases can be excluded when there is no sign of hepatic disease and fibrinogen levels and liver function are normal. DIC can also be excluded based on clinical manifestations, especially in severe cases in which clinical differentiation is possible, as this condition is always accompanied by thrombocytopenia and hypofibrinogenemia [13–16].

Vitamin K deficiency can occur as secondary deficiency after the neonatal period due to a lack of oral-intake vitamin K, alteration in gut flora as a consequence of long-term use of broad-spectrum antibiotics, liver disease, or malabsorption of vitamin K [13–16]. In this study, the VKDB cases were classical and late-onset VKDB variants. Late-onset VKDB is commonly accompanied by intracranial bleeding and severe anemia and is associated with a high mortality rate [20, 21]. VKDB contributes to neonatal mortality and morbidity. The case fatality rate of VKDB cases is high, as reported by Cekinmez et al., who found a survival rate of VKDB in infants of 50% with all deaths presenting intracranial bleeding [21]. The incidence rate of VKDB in Asia ranges from 20 to 116 per 100,000 births [22]. Takahashi et al. estimated the incidence of late VKDB in Japan to be 1.9 cases per 100.000 births [23]. This number is higher in developing countries than in developed countries [14, 22]. Despite the WHO recommendation, vitamin K prophylactic treatment in low-resource settings remains low [14, 24].

All VKDB cases in this study were determined to be coincidental events with a temporal association with immunization. VKDB was not considered to be caused by vaccine products, immunization error, or immunization anxiety [5]. This finding is in line with a previous study in West Java, Indonesia by Susanah et al.*,* who reported that VKDB is the main cause of severe bleeding in AEFI cases in children, with all cases categorized as coincidental events following immunization [9].

Vaccines are usually administered to healthy children. In this study, no subjects had a history of antibiotic use. There is still the possibility of other vitamin K deficiency risk factors due to the fact that all the subjects were exclusively breast-fed infants. The breast-fed infant has access to few sources of vitamin K, as it is transmitted poorly across the placenta and is present at low concentrations in human milk [25]. The catastrophic consequences of vitamin K deficiency are preventable with the administration of prophylactic vitamin K [14, 24–28]. Further studies to identify risk factors are required to formulate more advanced prevention programs and policies.

## Conclusion

Bleeding manifestation is sometimes reported as an AEFI. Most cases were found to be examples of VKDB, and were found to be coincidental events following immunization with a temporal association and belong to an unrelated category based on the results of a causality assessment. This study reinforces the recommendation of prophylactic vitamin K administration for all newborns and suggests that the Indonesian AEFI surveillance system should be improved to reduce the possibility of under-reporting.

## Data Availability

All data and materials of this study are available at West Java Provincial Committee of AEFI, Regional Health Office of West Java Province–Dr. Hasan Sadikin General Hospital/. Faculty of Medicine, Universitas Padjadjaran, Bandung, Indonesia, by contacting the corresponding author (Susi Susanah, E-mail: susi_rshs@yahoo.co.id) on reasonable request.

## Abbreviations

AEFI: Adverse event of following immunization
APCD: Acquired prothrombin complex deficiency
aPTT: Activated partial thromboplastin time
DIC: Disseminated intravascular coagulation
EPI: Expanded program immunization
Hep B_0_: Hepatitis B_0_
ITP: Immune thrombocytopenia purpura
PIVKA: Proteins induced by vitamin K absence
PT: Prothrombin time
TT: Thrombin time
VKDB: Vitamin K deficiency bleeding
